# Investigation of Renal Tissue Deposition of the Calcineurin Inhibitors Voclosporin, Cyclosporine and Tacrolimus Using MALDI-MSI Imaging

**DOI:** 10.1007/s11095-025-03943-y

**Published:** 2026-01-08

**Authors:** Simon Zhou, Krishani Kumari Rajanayake, Miao He, Bo Wen, Ankhbayar Lkhagva, Ernie Yap, Duxin Sun, Jennifer Cross, Kory Engelke, Robert B. Huizinga

**Affiliations:** 1 Aurinia Pharmaceuticals Inc, Edmonton, AB Canada; 2 University of Michigan, Ann Arbor, MI USA; 3 Reformation Consulting Services Inc, Victoria, BC Canada; 4 Aurinia Pharmaceuticals Inc, Rockville, MD USA

**Keywords:** active renal secretion, proximal tubule reabsorption, renal drug disposition, renal drug retention, selective drug uptake in kidney

## Abstract

**Background:**

Calcineurin inhibitors (CNIs) are immunosuppressive agents that inhibit calcineurin (CN) and are recommended for the treatment of lupus nephritis (LN). In clinical trials, differences in the safety profiles of CNIs have been observed. Emerging data suggests that small molecule therapeutics may be differentially distributed and retained within organ tissues, potentially explaining these safety profile disparities.

**Methods:**

This study investigated the renal distribution and retention of the CNIs voclosporin (VCS), tacrolimus (TAC), and cyclosporine A (CSA) in CD-1 mice using matrix-assisted laser desorption/ionization imaging mass spectrometry (MALDI-MSI).

**Results:**

Distinct patterns of the distribution and retention of these compounds were observed. VCS showed a moderate cortical distribution, peaking between 15- and 30 min post administration, and was then rapidly excreted with minimal renal retention observed by 2 h post-dosing. In contrast, CSA exhibited a diffuse, persistent distribution in renal structures for up to 4 h post-dosing. TAC showed a diffuse distribution pattern, with retention observed in the renal medulla for up to 7 h post-dosing.

**Conclusions:**

These data indicate that CNIs display different renal handling profiles. The shorter duration of renal retention of VCS demonstrated in the healthy mice indicates a differentiated profile compared to the other CNIs. Further research on the body-wide tissue distribution and renal handling of TAC, VCS and CSA in humans will aid in delineating the distinct clinical profiles of CNIs and optimize their use in treating immune disorders.

**Supplementary Information:**

The online version contains supplementary material available at 10.1007/s11095-025-03943-y.

## Introduction

Calcineurin inhibitors (CNIs) are immunosuppressive agents that inhibit calcineurin (CN), resulting in a reduction in interleukin-2 (IL-2) production [1]. This leads to a selective and reversible inhibition of lymphocyte proliferation and T-cell cytokine production [2]. An additional action of the CNIs is the stabilization of the podocyte cytoskeletal structure in the glomeruli, via inhibition of the calcineurin-mediated dephosphorylation of synaptopodin [2, 3]. Further, CNIs induce vasoconstriction of afferent arterioles, thus lowering intra-glomerular pressure and reducing proteinuria [4].

First-generation CNIs such as cyclosporine A (CSA) and tacrolimus (TAC) have been used in immunosuppression regimens for solid organ transplantation for decades, with efficacy subsequently demonstrated in the treatment of multiple autoimmune diseases [5, 6]. Recent guidelines recommend the use of CNIs in the treatment of lupus nephritis (LN), a common yet serious complication of systemic lupus erythematosus (SLE) [4, 7–9]. Guidelines recommend that for active Class III/IV ± V patients where kidney function is not severely impaired, one option for first-line therapy is the use of glucocorticoids in combination with a CNI and mycophenolate mofetil (MMF) [8, 10]. Maintenance therapy for LN is recommended to continue for at least three years following complete response, and patients are advised to remain on the first-line therapy regimen [9]. However, the safety profiles of first-generation CNIs makes their long-term clinical use challenging. CSA and TAC have narrow therapeutic windows and complex, variable pharmacokinetic properties that necessitate the therapeutic drug monitoring of patients to ensure efficacy and safety [3, 11]. CNIs as a class are associated with safety concerns that include acute and chronic nephrotoxicity, hypertension, electrolyte disturbances, infections, hyperglycemia, and dyslipidemia [3, 5, 12]. The prolonged use of high-dose, first-generation CNIs in solid organ transplants is implicated in the development of irreversible, chronic neuropathological lesions [12–14].

**Fig. 1:**
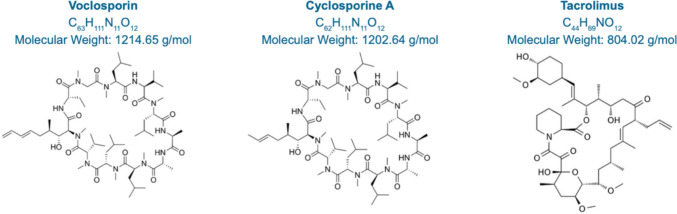
Chemical structures and properties of calcineurin inhibitors: Voclosporin, Cyclosporine A and Tacrolimus.

Voclosporin (VCS) is a second-generation oral CNI that is structurally similar to CSA with the addition of a functional group on the amino acid at position 1 (Fig. 1). This results in altered binding to CN and an increase in potency compared to CSA [15]. VCS has a linear and stable pharmacokinetic profile, in comparison to other CNIs, and therefore does not require therapeutic drug monitoring [2]. VCS is indicated for the treatment of patients with active LN, in combination with background immunosuppression [16].

Emerging evidence indicates that small molecule therapeutics display differential deposition within target and non-target organ tissues [17–19]. It is possible that CNIs are differentially distributed and retained in the kidney, which could explain the differences in their safety profiles demonstrated in clinical trials [20–24]. Matrix-assisted laser desorption/ionization imaging mass spectrometry (MALDI-MSI) is a technique that enables the tissue distribution of compounds to be studied, through the acquisition of mass spectra across the surface of a tissue section. A two-dimensional image of the compound’s ion intensity is overlaid across the tissue section, enabling the histology of the section to be compared to apparent compound distribution [17].

The aim of this investigation was to examine the intrarenal distribution of CNIs in mice using MALDI-MSI.

## Materials and Methods

### Chemicals

Chemicals and solvents (analytical grade) were purchased from the following sources: VCS (Aurinia Pharmaceuticals Inc, Canada), TAC (Sigma-Aldrich, USA), and CSA (Cayman Chemical, USA). LC − MS-grade acetonitrile, ethanol, and water, and trifluoroacetic acid (TFA) were purchased from Fisher Scientific. Chromophor EL and α-Cyano-4-hydroxycinnamic acid (CHCA, MALDI MS grade) were purchased from Sigma-Aldrich, USA. All chemicals used in this study were stored, handled, and disposed of according to local guidelines.

### Sample Preparation

All *in vivo* studies were performed under animal protocol PRO00011365, approved by the Institutional Animal Care and Use Committee (IACUC) of the University of Michigan, in accordance with the recommendations in the Guide for the Care and Use of Laboratory Animals of the National Institutes of Health. Female CD-1® IGS mice (strain code: 022, 6–8 weeks old) were purchased from Charles River Laboratories, USA. When collecting tissue samples for MALDI imaging, testing compounds were prepared at a concentration of 25 mg/mL in Chromophor EL and ethanol 1:1 v/v and were diluted to 6 mg/mL with saline. A single IV dosing at 30 mg/kg of each testing compound was administered to three animals per time point, and kidneys were harvested at 15 min, 30 min, 1 h, 2-h, 4-h and 7-h time points. All mice were euthanized for tissue collection with isoflurane. Kidney samples were extensively rinsed with phosphate-buffered saline (pH 7.4), then flash frozen in liquid nitrogen and stored at −20°C until sectioning. Ten micrometre kidney tissue sections from the middle of the left kidney were mounted on indium tin oxide (ITO) coated glass slides. A matrix preparation of 10 mg/mL CHCA in 85% acetonitrile/13% ethanol + 2% water + 0.1% TFA was formulated and sprayed onto the tissue sections using the HTX tissue sprayer (HTX Technologies LLC, USA), flow rate 0.2 mL/min, velocity 800 mm/min, drying time 2 s, line spacing 2.5 mm, sprayer temperature 80°C. The tissue section glass slides with CHCA matrix were dried for 10 min in a vacuum desiccator and subjected to MALDI-MSI using the AP-MALDI source (MassTech, USA) coupled to Thermo Orbitrap-IDX (Thermo Fisher Scientific, USA). Three replicate kidney images were obtained per time point for each of the three dosed animals.

### AP-MALDI-MSI

For imaging, Target-ng software (MassTech, USA) was used to control the XY stage motion and operation of the laser on AP-MALDI source. The source utilized a diode-pumped solid-state laser (λ = 355 nm) operating in “Constant Speed Raster” motion mode under 5 kHz repetition rate. Maximum laser pulse energy was 3 μJ at 1 kHz repetition rate. A beam attenuator was used to adjust laser energy. The kidney tissue sections were scanned with 30 μm laser size at 40 μm stepping size on AP-MALDI source. Mass spectra were acquired in a positive-ion mode with mass resolution up to 120,000 at m/z 400, and mass range of m/z 800 − 1300 on Orbitrap-IDX. The mass spectrometry imaging was developed using the potassium adduct of each test compound, CSA at m/z of 1240.8051, VCS at m/z of 1252.8051, and TAC at m/z of 842.4457 within 5 ppm accuracy. An automatic gain control (AGC) target was set to 5 × 10^6^ with 200 ms maximum injection time. Data analysis and visualization were performed with Thermo Xcalibur 4.4 (Thermo Fisher Scientific, Waltham, MA) and SCiLS Lab MVS software (Bruker, USA).

## Results

**Fig. 2:**
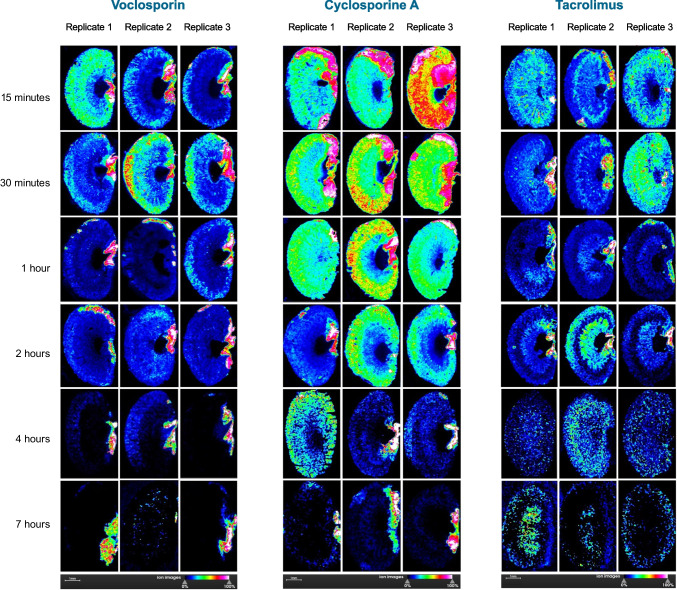
MALDI-MSI imaging of mouse CD-1 kidneys. Each panel represents the imaged kidney from a single animal, with three replicates imaged at each time point for each calcineurin inhibitor. The greater the concentration of drug present, the greater the intensity of colour in the image, with blue indicative of low concentrations and from green to red indicative of increasing concentrations (note the ion images scale beneath each panel). VCS = voclosporin, CSA = cyclosporine A and TAC = tacrolimus.

MALDI-MSI imaging revealed a differential pattern of distribution and retention of VCS, TAC, and CSA in healthy CD-1 mouse kidneys (Fig. 2). Supplementary Fig. 1 shows a MALDI-MSI image of a control CD-1 mouse kidney. VCS demonstrated an apparent moderate cortical distribution that peaked between 15- and 30 min post-dosing and then dissipated via rapid excretion. By two hours post-dosing, low concentrations of VCS in the cortex remained. By seven hours post-dosing, minimal VCS was detectable in cortical and medullary structures, and minimal VCS was detected in the location of the ureter at this time point. In contrast, CSA was detected in higher concentrations than VCS throughout renal structures from 15 min to 1-h post-dosing. CSA demonstrated a longer retention in the cortex than VCS, with moderate concentrations remaining at four hours post-dosing. By seven hours post-dosing, minimal CSA was detected in renal structures, and residual CSA was also detected in a ureteral location. TAC was detected in a diffuse pattern from 15 min post-dosing and appeared to show higher retention than VCS but lower retention than CSA in both cortical and medullary structures. TAC appeared to demonstrate prolonged retention in medullary structures, with moderate concentrations remaining at 7 h post-dosing.

## Discussion

This study has revealed a differential pattern of distribution and retention of VCS, CSA, and TAC in CD-1 mouse kidneys. Specifically, moderate cortical distribution of VCS was observed compared to the more diffuse distribution of CSA and TAC. The intrarenal retention of VCS was minimal. By two hours post-drug administration, only a low concentration of VCS could be detected. In contrast, the concentration of CSA remained moderate for up to four hours post-dosing and a moderate concentration of TAC was detected in the medulla for over seven hours post-dosing. Therefore, the data obtained by MALDI imaging in mice suggests that CSA and TAC are retained in the kidneys, potentially via tubular reabsorption, while VCS is secreted into the filtrate. These findings are consistent with their respective renal clearance, reabsorption, and active secretion in humans (Table I). In healthy human subjects, CSA has a renal clearance representing approximately 10% of expected passive filtration, and TAC has a renal clearance representing < 2% of expected passive filtration. In comparison, VCS has increased renal clearance, approximately 200% of its expected passive filtration rate. High renal clearance with respect to passive filtration is indicative of a component of active secretion [25–27]. Although VCS is structurally different from CSA by only one methyl group at amino acid −1 position (Fig. 1), this minor structural modification may change its interaction with albumin and other proteins in organ tissues. For example, the substitution of a single hydroxyl to an amino group changes iophenoxic acid to iopanoic acid and reduces the high-affinity binding of iophenoxic acid to human albumin from a 2.5 year half-life to the relatively low-affinity binding of iopanoic acid to approximately two weeks [28]. Research into the binding of VCS and CSA to human albumin may yield further insights into their respective tissue disposition.

**Table I:** Renal Clearance, Reabsorption and Secretion Data of CNIs in Humans

	CL (mL/min)	CL/F (mL/min)	fu (%)	Expected CLr (mL/min)	Actual CLr (mL/min)
Cyclosporine A	210–240	500–600	10	12.5^1^	1.48
Tacrolimus	37.5	N/A	1	1.25^2^	0.014
Voclosporin*	N/A	1060	3	3.75^3^	7.82

*Pharmacokinetic data for voclosporin specific to the orally administered softgel formulation. Expected CLr > actual CL is suggestive of renal reabsorption. Expected CLr < actual CL is suggestive of renal secretion

*CL* Clearance; *CLr* Renal clearance; *CL/F* Apparent clearance; *CNIs* Calcineurin inhibitors; *fu* fraction unbound in plasma; *F* Bioavailability; *GFR* Glomerular filtration rate; *N/A* Not applicable

Expected CLr values are estimated using the formula GFR x fu and are based on the following reference documents

^1:^Ptachcinski R *et al*. Clin Pharmacokinet. 1986;11(2):107–32 [29]

^2:^Möller A *et al*. Drug Metab Dispos 1999 Vol. 27 Issue 6 Pages 633–6 [30]

^3:^Mayo PR *et al*. J Clin Pharmacol. 2013;53(8):819–826 [31]; Aurinia Pharmaceuticals Inc. Data on File. 2020; NDA 213716: 2.7.2 Summary of Clinical Pharmacology Studies.)

While VCS, CSA, and TAC are all CNIs, they exhibit distinct pharmacokinetic and clinical safety profiles [2]. In terms of safety profile, CSA is associated with an increase in factors such as endothelin, resulting in a dose-dependent vasoconstriction of vessels and leading to acute kidney function impairment [32]. CSA has also been associated with chronic nephropathy, caused by direct toxic effects on tubular epithelial cells and hemodynamic changes, particularly when used in long-term renal transplant settings [32]. A similar nephrotoxicity is observed with TAC [13]. Additionally, TAC may cause electrolyte disturbances such as hyperkalemia and hypomagnesemia [5] and directly inhibits the secretion of insulin from human islet cells, leading to hyperglycemia [33]. CSA and TAC are associated with the development of hyperlipidemia in renal transplant patients in contrast to VCS, which is associated with favorable effects on lipid concentrations [34, 35]. Clinically, long-term TAC treatment, particularly in the renal transplant setting, is associated with an increased incidence of new-onset diabetes, while VCS has shown no significant impact on glucose metabolism [5, 13, 33, 35, 36]. Further, a drug-drug interaction exists between CSA and the active metabolite of MMF, whilst this has not been observed with VCS or TAC [15, 37]. In the AURORA clinical program for patients with active LN, treatment with VCS, MMF and low-dose glucocorticoids resulted in rapid, sustained reductions in proteinuria and demonstrated an acceptable long-term safety profile, [3, 35, 38, 39]. Additionally, there was no evidence of chronic kidney injury after up to three years of treatment in a recent AURORA 2 biopsy sub-study in LN patients [40]. Further, VCS has demonstrated no increased safety risks relating to hyperglycemia, hyperlipidemia or electrolyte disturbances [35, 38, 39].

It is possible that the differential pharmacokinetic disposition of VCS, CSA, and TAC in central and peripheral renal compartments may result in differences in systemic or off-target tissue drug exposure [2, 24]. These potential underlying differences in distribution within the renal structures, and the extent and duration of tissue exposure to drug, may help to explain the differences in the observed clinical profile of each CNI. This study may therefore contribute to the understanding of the mechanisms behind the different clinical outcomes of VCS treatment, compared to other CNIs. Due to the fast renal excretion of all three CNIs and limits of molecular excitation by MALDI, initial experiments at lower doses of 5 and 10 mg/kg resulted in inferior signal. Therefore, a relatively high dose of 30 mg/kg for each CNI was selected for this analysis. More studies at lower doses are required to fully understand this aspect of CNI function and further research with more sensitive multiplex immunostaining and imaging of distinct renal cell types is ongoing.

A limitation of this study is the different sensitivity of the MALDI technique to different compound properties. CSA and VCS are structurally very similar and less hydrophobic compared to TAC, allowing for more confidence in the interpretation of the comparative results from MALDI imaging for CSA and VCS. The hydrophobic nature of TAC molecules and other factors such as proton affinity and solvent compatibility affects the MALDI ionization and makes the interpretation of these results more challenging, as the intensity of luminescence may not directly represent the actual drug concentrations [41]. The use of MALDI-MSI for quantitative measurements requires further validation such as the incorporation of internal standards or calibration-based methods.

## Conclusion

In conclusion, the observed renal retention and distribution of VCS in healthy mice may be associated with its improved safety profile compared to the more diffuse distribution and greater renal retention of CSA and TAC. Our findings further contribute to a growing body of evidence distinguishing the unique features of VCS from CSA and TAC [33], and may also have value in other diseases that are treated with calcineurin inhibition. Further research on the distribution and renal handling of TAC, VCS and CSA in humans will aid in delineating the distinct clinical profiles of CNIs and guide their optimal uses to treat immunological disorders.

## Supplementary Information

Below is the link to the electronic supplementary material.

Supplementary file1 (DOCX 313 KB)

## Data Availability

Data will be shared with researchers on reasonable request to the corresponding author. Data will be shared through a secure online platform after signing a data access agreement.
