# Supplementary material for: Investigation of Renal Tissue Deposition of the Calcineurin Inhibitors Voclosporin, Cyclosporine and Tacrolimus Using MALDI-MSI Imaging
Source: Pharm Res. 2026 Jan 8;43(1):149–55. doi: 10.1007/s11095-025-03943-y (PMC12913290; doi:10.1007/s11095-025-03943-y)
Supplement: Supplementary file 1 — Supplementary file1 (DOCX 313 KB) [file 11095_2025_3943_MOESM1_ESM.docx]

Supplementary Figure 1: MALDI-MSI image of control CD-1 mouse kidney tissue


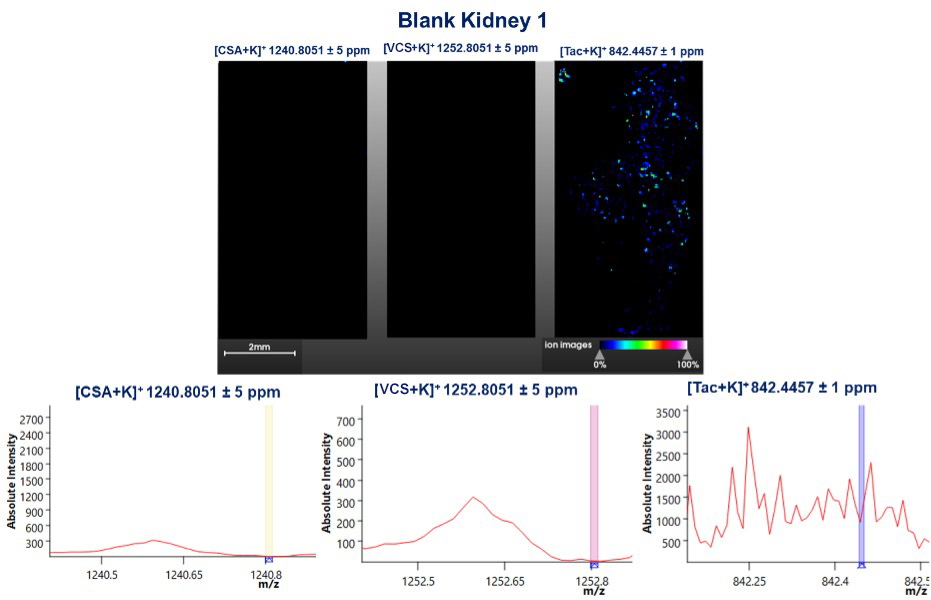


MALDI-MSI imaging of mouse negative control CD1 kidney. Each panel represents the imaged kidney from a single animal. The greater the concentration of drug present, the greater the intensity of colour in the image, with blue indicative of low concentrations and from green to red indicative of increasing concentrations (note the ion images scale beneath the panel). The respective mass of CSA, VCS and TAC are shown in the panel below.
